# Macrocyclic bis(ureas) as ligands for anion complexation

**DOI:** 10.3762/bjoc.10.193

**Published:** 2014-08-12

**Authors:** Claudia Kretschmer, Gertrud Dittmann, Johannes Beck

**Affiliations:** 1 Institute for Inorganic Chemistry, University of Bonn, Gerhard-Domagk-Str. 1, 53121 Bonn, Germany

**Keywords:** anion binding, macrocyclic compounds, NMR spectra, supramolecular chemistry, template, urea

## Abstract

Two macrocyclic bis(ureas) **1** and **2**, both based on diphenylurea, have been synthesized. Compound **1** represents the smaller ring with two ethynylene groups as linkers and **2** the larger ring with two butadiynylene groups. On thermal treatment to 130 °C molecule **1** splits up into two dihydroindoloquinolinone (**3**) molecules. Both compounds **1** and **2** form adducts with polar molecules such as dimethyl sulfoxide (DMSO) and dimethylformamide (DMF) and act as complexing agents towards a series of anions (Cl^−^, Br^−^, I^−^, NO_3_^−^, HSO_4_^−^). The crystal structures of **3**, **2**·2DMSO, **2**·2DMF, and of the complex NEt_4_[Br·**2**] have been determined. Quantitative investigations of the complexation equilibria were performed via ^1^H NMR titrations. While **1** is a rather weak complexing agent, the large ring of **2** binds anions with association constants up to log *K* = 7.93 for chloride ions.

## Introduction

Supramolecular chemistry – the “chemistry beyond the molecule” – is an area of modern chemistry, which has grown exponentially in the last decades [1–2]. Among the many concepts of supramolecular interactions, anion coordination chemistry has always been an intensively explored field, which still offers substantial progress [3–5].

The urea group has proven to be an excellent anion receptor. The ability to form two directional hydrogen bonds through the highly polarized N−H groups towards different kinds of anions allows for building particular molecular arrangements. Incorporation of two or three urea groups into one molecular entity enables the formation of complex building units. Tripodal tris(ureas) were synthesized, which contain tetrahedral oxoanions like phosphate or sulfate [6–7] and ligands with three concatenated urea functions were shown to form 2:2 complexes with phosphate anions [8]. Anion complexation is also possible with a rigid planar ligand, as was shown by indole-based macrocycles [9]. This ligand system represents a stiff ring of two bis(indole) units connected via two ethynylene groups as linkers. As proven by a crystal structure determination, a chloride ion fits into the cavity bound via four N−H∙∙∙Cl bonds and strong complexation was observed with several other anions. In the ^1^H NMR spectra a significant dependence of the chemical shift of the N−H proton with the kind of bound anion was observed and the association constants as high as log *K* = 6.2 for Cl^−^ were determined.

We achieved the synthesis of a macrocyclic planar bis(triazene), in which two diphenyltriazene units were linked by two ethynylene groups. On deprotonation, a dianionic planar bis(triazenide) ligand is formed, which takes up several different transition metal ions, preferredly in the divalent state [10]. Linking two diphenylurea groups by one ethynylene or butadiynylene group gives a stiff arrangement but leaves one degree of freedom in the system, since rotation around the linking group is possible. This approach was realized by Steed and coworkers, who showed that on adding chloride ions planarization occurs and a rather high binding constant of log *K* = 2.55 for the complexation of Cl^−^ was determined [11]. Introducing a second bridging unit would give a ring containing two diphenylurea units connected via two stiff linking units. Since the urea unit N−C(O)−N and the triazenide unit N−N=N are isosteric, we complemented our bis(triazene) ligand system by cyclic bis(ureas). This opens the possibility for complexation of cations and anions with two isosteric ligands, just under exchange of the active groups within the respective ring system. Here we describe our results concerning macrocyclic bis(ureas) with a rather rigid molecular entity.

## Results and Discussion

**Scheme 1:**
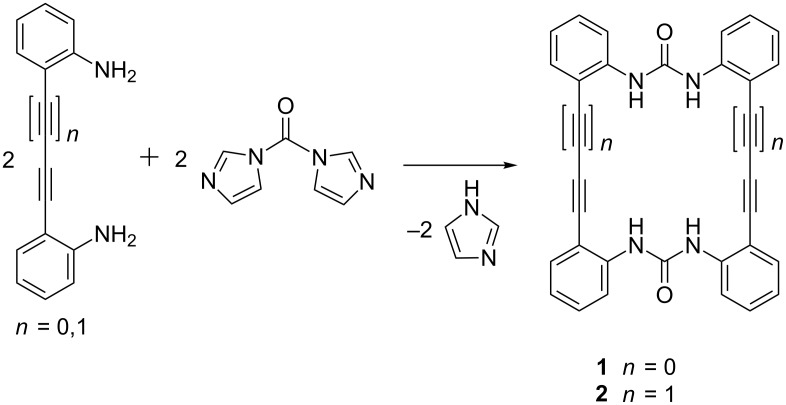
Synthesis of the macrocyclic bis(ureas) **1** and **2**.

The synthesis of the two macrocyclic diphenylureas **1** and **2** proceeds straightforward from the respective 2,2’-diamino derivatives of diphenylethyne (tolane) and diphenylbuta-1,3-diyne with carbonyldiimidazole (Scheme 1).

**Scheme 2:**
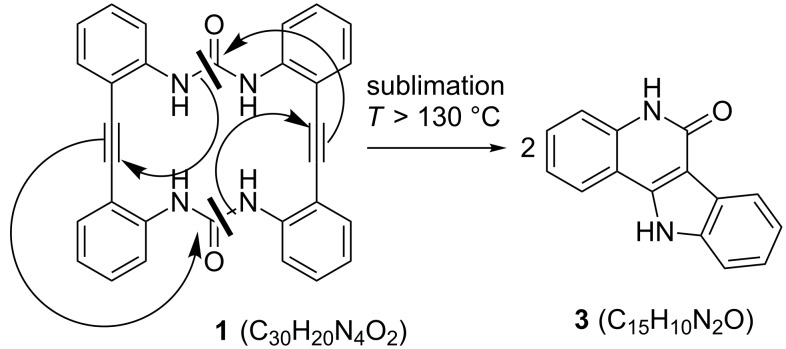
Formation of dihydroindoloquinolinone **3** from **1** by vacuum sublimation.

In both cases, the cyclization products were selectively formed in good yields. The quest for the molecular conformation is challenging for both molecules. Since the bis(triazenide) congeners of **1** and **2** are planar, one can presume an analogous molecular shape for the bis(ureas). All attempts to obtain single crystals of **1** for a structure determination failed. Crystallization from solution gave only microcrystalline material. When we tried to obtain crystals via vacuum sublimation, a complete vaporisation at temperatures above 130 °C and deposition of colourless crystals were observed. A crystal structure analysis of the deposited crystals, however, revealed that a fragmentation and rearrangement reaction had occurred. Under the applied conditions **1** is completely converted to dihydroindoloquinolinone **3** (Scheme 2).

In accordance with the effortlessness of the conversion reaction, the mass spectrum of **1** is dominated by the [M/2]^+^ signal. The indoloquinolinone **3** forms essentially planar molecules (see Supporting Information File 1, Figure S8). Indoloquinolinones can be synthesized by multistep procedures from suitable precursors [12–14]. Using thermolysis for the synthesis of indoloquinolines has already been reported. Cyclization of aminophenyl substituted tolane isocyanates lead to indoloquinolinones. The crystal structure of the *N*-methylated congener of **3** has been determined [15].

Thermal treatment of **2** does not lead to fragmentation or sublimation of volatile material as observed for **1**. Macrocycle **2** is soluble in dimethylformamide (DMF) or dimethyl sulfoxide (DMSO) and can be crystallized from solution as light-yellow crystals (see Supporting Information File 1, Figure S4). These crystals incorporate associated solvent molecules, which are difficult to remove and lead to discrepancies in the elemental analyses towards the calculated compositions even after excessive pumping at elevated temperatures. A bluish coloration of the yellow material is already observed after prolonged keeping under vacuum at ambient temperature. Thermal treatment in vacuo at temperatures above 200 °C leaves a dark blue material (see Supporting Information File 1, Figure S2). Dissolving the blue material in DMF or DMSO leads to an almost complete dissolution and a yellow solution, leaving behind only a small portion of dark insoluble material. The nature of the yellow-to-blue transformation is presently unclear. Partial cyclization reactions in the solid material or formation of polymers via radical mechanisms seem probable.

**Figure 1:**
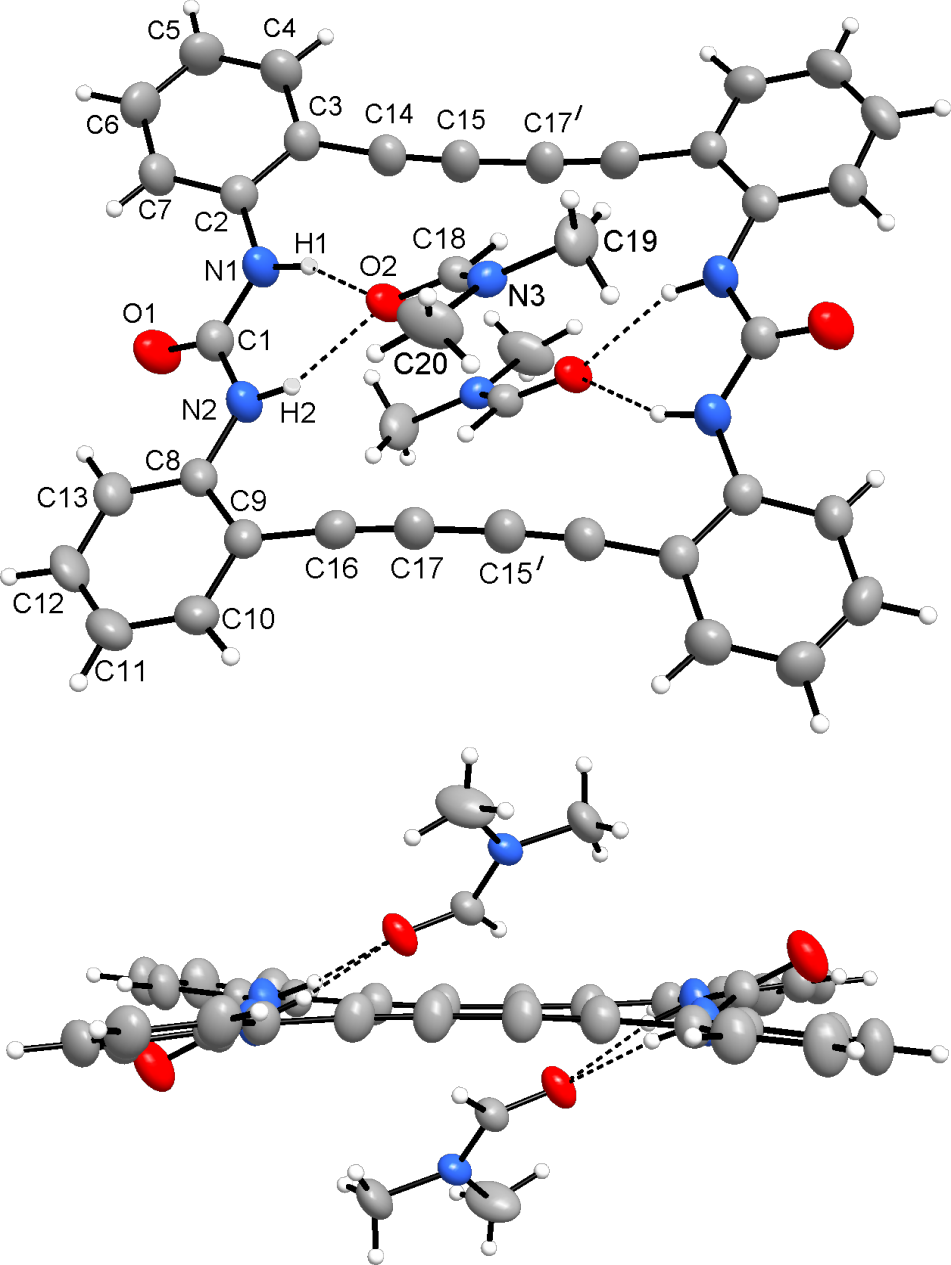
The molecular structure of **2**·2DMF in two different views, on top perpendicular to the plane, on bottom in the plane of the macrocycle. The molecular complex bears an inversion centre in the midpoint. Thermal ellipsoids are scaled at the 50% probability level. For a figure of the molecular structure of **2**·2DMSO see Supporting Information File 1, Figure S9.

When crystallized from DMF or DMSO, **2** forms stable 1:2 adducts with these solvent molecules. Crystals were examined by X-ray single crystal diffraction [16]. Both compounds, **2**·2DMF and **2**·2DMSO, are not crystallographically isotypic but the molecular entities are completely analogous and may be discussed jointly. In both cases the macrocyclic ring is mainly flattened and two molecules of DMF or DMSO are coordinated above and below the ring plane (Figure 1). A plane through all atoms except the C and O atoms of the urea groups is rather well fulfilled with the largest deviation found for C13 with 0.3 Å. The urea groups themselves are planar but tilted by 28° against the main plane of the outer ring. This is caused by the N−H∙∙∙O bonds to the O atom of the DMF molecule with H∙∙∙O separations of 2.00(1) and 2.07(2) Å. The somewhat lower basicity of DMSO is manifested in longer N−H∙∙∙O bonds of 2.09(1) and 2.18(2) Å in the **2**·2DMSO adduct.

Since immediate experimental data for the structure of the unsolvated macrocyclic bis(ureas) were not obtainable, we used molecular mechanics calculations as implemented in the SPARTAN program suite [17] to calculate the respective structures. In both cases, the molecules are obtained as far from planarity (see Supporting Information File 1, Figure S6 and Figure S7). Actually, a strong tilting is expected for both **1** and **2**. According to these calculations, the small ring of **1** forces the two urea groups into a head-to-tail arrangement with short intramolecular hydrogen bridges. An even stronger tilting of the molecule is expected for **2**. Here, the distances are too large for any intramolecular N−H∙∙∙O bridging bonds.

**Figure 2:**
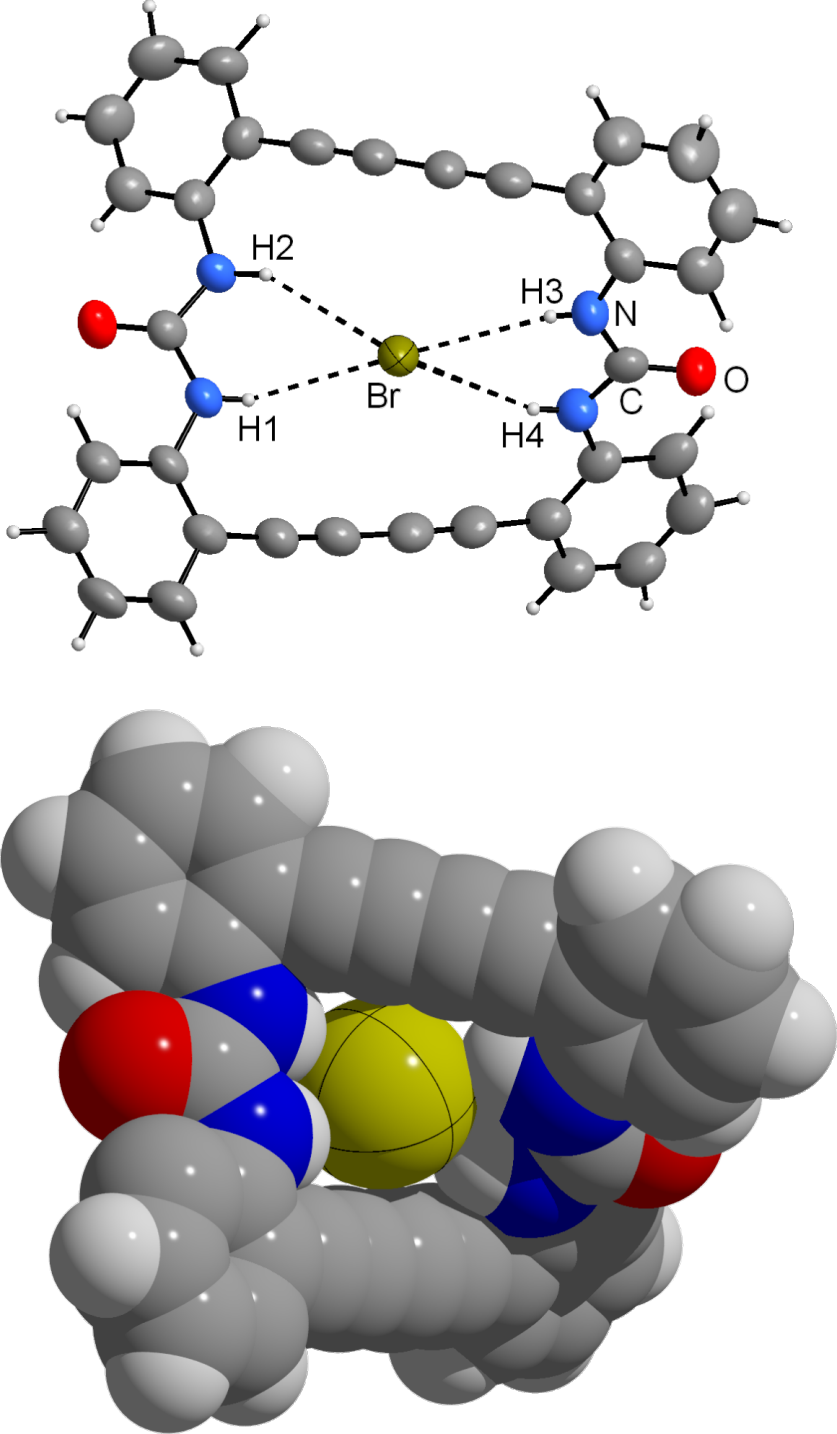
Molecular structure of the anionic complex in NEt_4_[Br·**2**]. Two different representations are given, on top with thermal ellipsoids scaled at the 50% probability level, on bottom in a space filling representation showing the strong twist of the macrocyclic bis(urea) ligand.

As a strong anion complexing agent **2** binds halide anions even in DMSO as solvent, despite DMSO itself is bound to the urea functional groups (Figure 1). If an excess of NEt_4_Br is added to a solution of **2** in DMSO, on slow evaporation yellow crystals of NEt_4_[Br·**2**] are separated. The crystal structure consists of ion pairs, tetraethylammonium cations and bromide anions, which are located in the cavity of the macrocycle (Figure 2). The ligand is strongly tilted. The four N−H∙∙∙Br bonds, however, show uniform lengths (H1−Br, 2.72; H2−Br, 2.74; H3−Br, 2.75; H4−Br, 2.71 Å; N−H∙∙∙O angles 149–169°). The representation with space filling radii shows that the halide anion fits well into the bis(urea) ring. The ammonium ions are located in the saddle shaped cavity formed by the twisted bis(urea).

**Figure 3:**
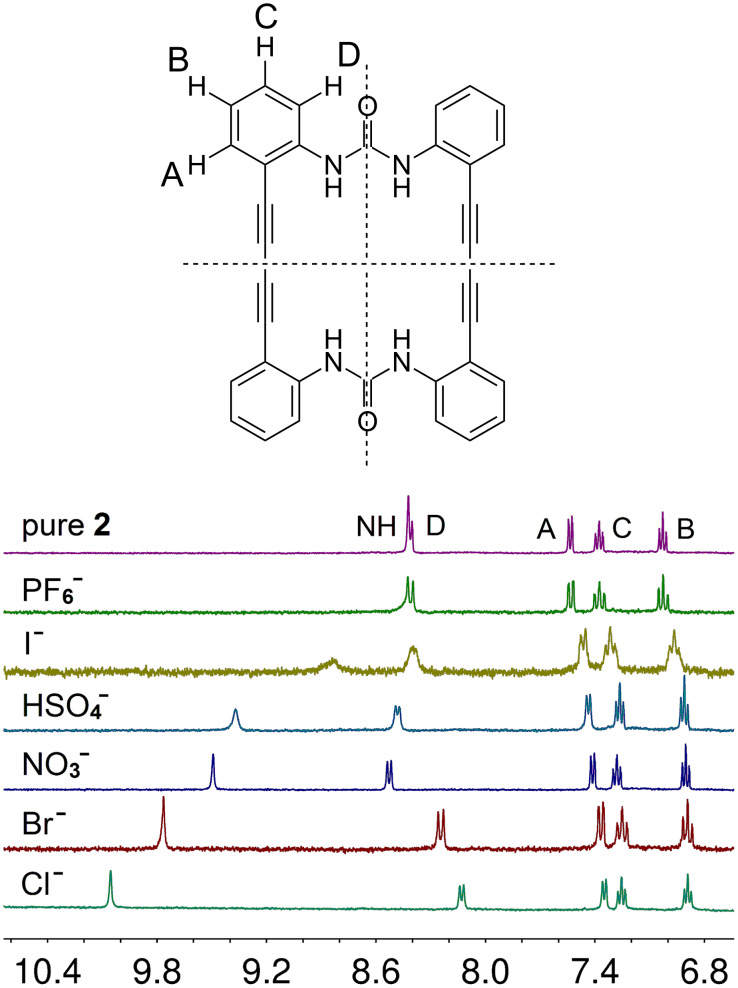
^1^H NMR spectra of **2** in THF-*d*_8_ after addition of several different tetrabutylammonium salts. The N−H proton resonances show a distinct dependence on the kind of the present anion.

The binding properties of **1** and **2** towards anions were studied by ^1^H NMR spectroscopy. As **2** was sufficiently soluble, tetrahydrofuran (THF) turned out as a suitable solvent for the spectroscopic investigations. On addition of tetrabutylammonium salts with different anions significant changes in the spectra emerge. The N−H proton resonances are shifted downfield with increasing effect in the order of I^−^ < HSO_4_^−^ < NO_3_^−^ < Br^−^ < Cl^−^. With the large complex anion PF_6_^−^, however, no effect was detected, indicating that no interaction occurred. The phenyl proton resonances are also affected by the effect. The protons at positions A, B, C (see Figure 3) are shifted slightly upfield, while the phenyl protons in position D *ortho* to the urea substituents are shifted downfield for the weaker complexes with HSO_4_^−^ and NO_3_^−^ but upfield for the stronger complexes formed with Br^−^ and Cl^−^. The effect on the phenyl protons amounts to maximally 0.3 ppm and is much weaker than the effect on the urea protons where shifts up to 1.7 ppm are observed. The relatively strong influence on the resonances of the ortho positioned protons may arise from the interaction with the carbonyl oxygen atom of the urea groups. This interaction is sensitive on the conformation of the flexible macrocyclic ring system. Comparing the crystal structures of **2**·2DMSO/DMF with [Br·**2**]^−^ shows a slight decrease of the mean O···H(ortho) distance from 2.32 to 2.25 Å.

For **1**, which is in pure form soluble only in DMF or DMSO, it was possible to record spectra in acetone-*d*_6_, since the solubility was highly increased by the addition of ammonium salts and subsequent complex formation. The effects on the N−H proton resonances are weaker compared with **2** (see Supporting Information File 1, Figure S11).

**Figure 4:**
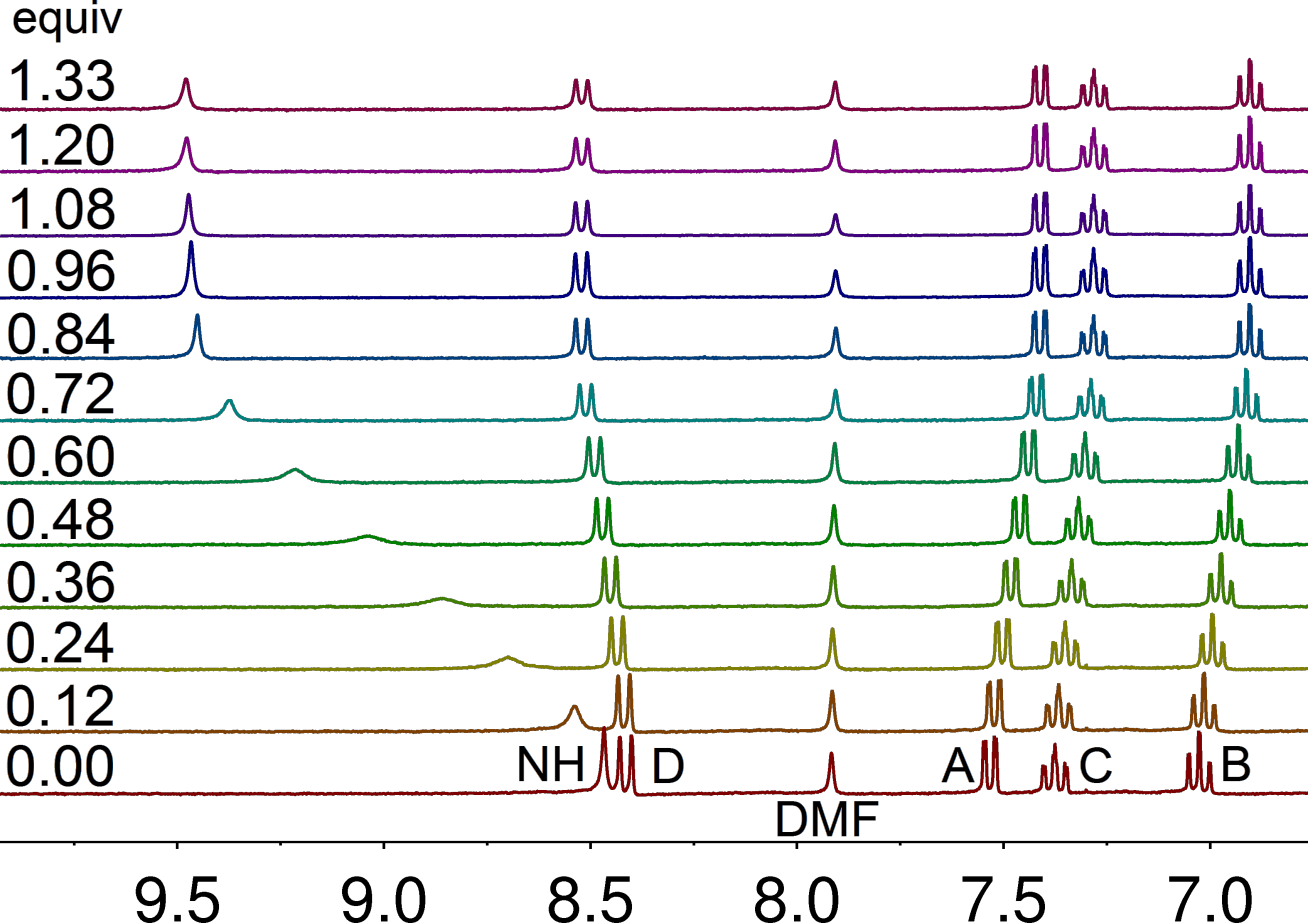
^1^H NMR spectra of **2** in THF-*d*_8_ after addition of increasing molar equivalents of tetrabutylammonium nitrate. The N−H proton resonance shows a distinct downfield shift depending on the concentration of the anion. The small invariant DMF signal originates from the solvent of recrystallization used to purify the sample for the NMR experiments.

Since no signals of the free host and of the anionic complexes are simultaneously present, the exchange rate between the anion and the host molecules is fast compared to the NMR timescale. However, the broadening of the signals by adding anion amounts around 0.5 equivalents may be interpreted as a coalescence phenomenon caused by an exchange between loaded and unloaded host. After the amount of the anion reaches a molar ratio of 1:1, the N−H proton resonance of **2** shows no further increase in the shift, indicating that a 1:1 complex has been formed (Figure 4). To ensure the composition of the host–guest complexes Job plots were used. The function molar fraction vs the product of molar fraction multiplied by the shift change Δδ allows for the determination of the molar fractions of host and guest. From the maximum of the extrapolated curve the molar fraction of the complex is obtained (see Supporting Information File 1). For I^−^, NO_3_^−^, and Br^−^ maxima at 0.50, 0.54, and 0.55 were found, which leads to the ratio of one host molecule and one guest molecule. For Cl^−^, the anion with the strongest shift effect, a maximum at 0.68 is present, indicating a complex of two bis(urea) molecules and one chloride ion. For the smaller macrocyclus **1** the Job plots do not show a distinct plateau after addition of one equivalent of anion. Here, doubtless an interaction is present but apparently not a distinct host–guest complex formation.

The binding constants were determined with the help of the program WinEQNMR2 [18]. Table 1 contains the obtained constants. The association constants of **2** with nitrate, bromide and iodide were obtained by fitting the titration curves and the subsequent calculation to a 1:1 binding mode. This binding mode was already confirmed by the Job plots for all anions except Cl^−^. Because the Job plot for the complex formation between **2** and nitrate shows the maximum of the curve not exactly at a molar ratio of 0.5 additional investigations via ESI mass spectrometry studies of NEt_4_[NO_3_·**2**] were undertaken. Signals at *m*/*z* = 515.2 and 578.2 clearly indicate the masses of the neat macrocycle **2** and the complex [NO_3_·**2**]. Signals originating from higher masses at 640.13 (**2** with two molecules nitrate) or 1094.31 (two molecules of **2** with one molecule nitrate) could not be detected. So the 1:1 binding mode seems the most plausible ratio. After evaluating the Job plots of the complex of **2** and chloride, a 2:1 binding mode for the calculation of the association constants was used. Reliable association constants for the smaller macrocycle **1** could only be obtained in the case of chloride.

**Table 1:** Binding constants of the macrocyclic bis(ureas) **1** and **2** towards different anions. All anions were used as tetrabutylammonium salts. Compound **2** was dissolved in THF-*d*_8_ and **1** in DMSO-*d*_6_. All NMR spectra were taken at room temperature (298 K).

Anion	Receptor **2**			Receptor **1**	
			
	*K*	log *K*		*K*	log *K*

I^−^	1775 ± 97 M^−1^	3.25			
NO_3_^−^	43.556 ± 430 M^−1^	4.64			
Br^−^	5.1∙10^6^ ± 5100 M^−1^	6.71			
Cl^−^	8.57∙10^7^ ± 9.21∙10^4^ M^−2^	7.93		202 M^−1^	2.31

A short look at the binding constants reflects the amount of the chemical shifts in the ^1^H NMR spectra. Bromide is bound strongly to **2** in the 1:1 binding mode. Nitrate is bound weaker than bromide but still stronger than iodide, and chloride in the 2:1 binding mode shows a high association constant of log *K* = 7.93. The binding constants for the known ring opened congener of **2** are significantly lower (log *K* (Cl^−^) = 2.55 and log *K* (Br^−^) = 1.56) [11] underlining the vast influence of the macrocyclic effect.

## Conclusion

Two macrocyclic bis(ureas) have been synthesized and examined for their anion complexation properties. The larger ring with two butadiynylene groups as spacers turned out as a suitable receptor for small anions with high binding constants. The molecule is flexible. In the complex with a bromide ion embedded in the ring cavity, the cyclic ligand is strongly tilted. The smaller cycle with ethynylene groups as linkers is a much weaker anion complexing agent.

## Experimental

The procedures for the syntheses of **1**, **2**, and **3** and the spectroscopic characterizations are described in detail in Supporting Information File 1.

## Supporting Information

File 1 Detailed experimental procedures, details of the crystal structure determinations and spectroscopic data for **1**, **2**, and **3**.

File 2 X-ray crystallographic data for **2**·2DMF, **2**·2DMSO, NEt_4_[Br·**2**], **3**, CCDC 993209, CCDC 993210, CCDC 993207, CCDC 993208.
