# Supplementary material for: Macrocyclic bis(ureas) as ligands for anion complexation
Source: Beilstein J Org Chem. 2014 Aug 12;10:1834–9. doi: 10.3762/bjoc.10.193 (PMC4142877; doi:10.3762/bjoc.10.193)
Supplement: File 1 — Detailed experimental procedures, details of the crystal structure determinations and spectroscopic data for 1, 2, and 3. [file Beilstein_J_Org_Chem-10-1834-s001.pdf]

Supporting Information

for

Macrocyclic bis(ureas) as ligands for anion complexation

Claudia Kretschmer[1], Gertrud Dittmann[1] and Johannes Beck*[1]

Address: [1]Institute for Inorganic Chemistry, University of Bonn, Gerhard-Domagk-Str. 1, 53121 Bonn, Germany

Email: Johannes Beck - j.beck@uni-bonn.de

* Corresponding author

Detailed experimental procedures, details of the crystal structure determinations and spectroscopic data for 1, 2, and 3

Contents

Experimental

Crystallography

NMR Spectra

Experimental

1. Synthesis of *cyclo*-bis(ethynyldiphenylurea) (**1)**

a) Synthesis of 2-nitrobenzalchloride

The synthesis was performed according to A. Kliegl, *Ber. Deutsch. Chem. Gesell*. **1907**, *40*, 4937-4941.

b) Synthesis of 2,2'-dinitrotolane

The synthesis was performed according to A. Kliegl, *Ber. Deutsch. Chem. Gesell*. **1911**, *44*, 1209-1218.

c) Synthesis of 2,2'-diaminotolane

The synthesis was performed according to P. Knops, F. Vögtle, *Chem. Ber*. **1991**, *124*, 1223-1227.

d) Synthesis of *cyclo*-bis(ethynyldiphenylurea) **1**

In a 250 mL round flask 0.470 g (2.26 mmol, 1 equiv.) of 2,2'-diaminotolane were dissolved in 70 mL dry THF. 1.098 g (6.77 mmol, 3 equiv.) 1,1'-carbonyldiimidazole were added and the mixture was heated under reflux for 7 h. After cooling to ambient temperature, the solvent was distilled off and the residue was treated with ethyl acetate. After filtration, the organic phase was washed with diluted hydrochloric acid and water and finally dried with anhydrous Na_2SO_4. Evaporation of the solvent yielded 0.32 g (60 %) of a white powder of mp. 310 - 313 ºC.

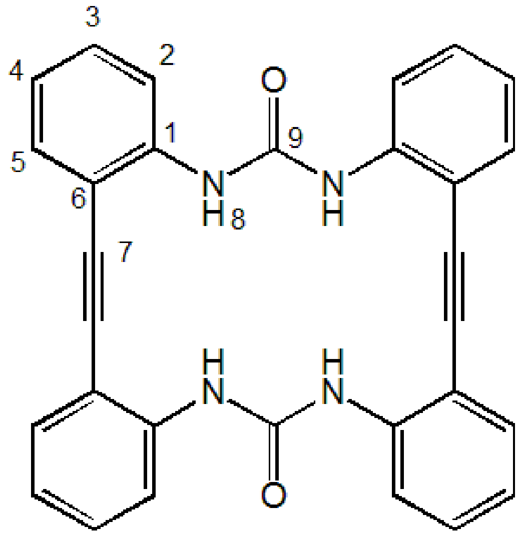

Formula and atom numbering of the cyclic bis(ethynyldiphenylurea) **1**

^{1}H-NMR (DMSO-d_6) δ/ppm: 9.14 (s, 1H, NH), 8.3 (d, 1H, CH(2), $^{3}J_{HH}$ = 8,46 Hz); 7.63 (dd, 1H, CH(5), $^{3}J_{HH}$ = 7,77 Hz, $^{1}J_{HH}$ = 1,65 Hz); 7.37 (dt, 1H, CH(3), $^{3}J_{HH}$ = 7,38 Hz, $^{1}J_{HH}$ = 1,66 Hz); 7.07 (t, 1H, CH(4), $^{3}J_{HH}$ = 7,53 Hz)

^{13}C-NMR (DMSO-d_6) δ/ppm: 152,13 (C9); 139,19 (C4); 133,90 (C1); 129,53 (C2); 122,26 (C5); 119,55 (C6); 111.36 (C3); 91,99 (C7, C≡C)

Mass spectrum (EI, inlet temperature 280 ºC, m/z (Int.)): 468.2 (8) $[M]^{+}$; 452.1 (3) $[M\text{-}O]^{+}$; 426.1 (1,5) $[M\text{-}N_2O]^{+}$; 336.1 (5) $[M\text{-}C_7H_4N_2O]^{+}$; 320.1 (8) $[C_{23}H_{16}N_2]^{+}$; 276.0 (3) $[C_{16}H_{11}N_3O_2]^{+}$; 260.0 (35) $[C_{16}H_{10}N_3O]^{+}$; 234.1 (100) $[M/2]^{+}$; 208.1 (40); 180.1 (10); 152.0 (8); 104.0(5)

Analysis: $C_{30}H_{20}N_4O_2$ (M_{rv}= 468.51 gmol^{-1}) calc. 76.91 C, 4.3 H, 11.95 N, 6.84 O; found 74.1 C, 4.7 H, 11.45 N.

Fig. S1

^{1}H-NMR spectrum of **1** in DMSO

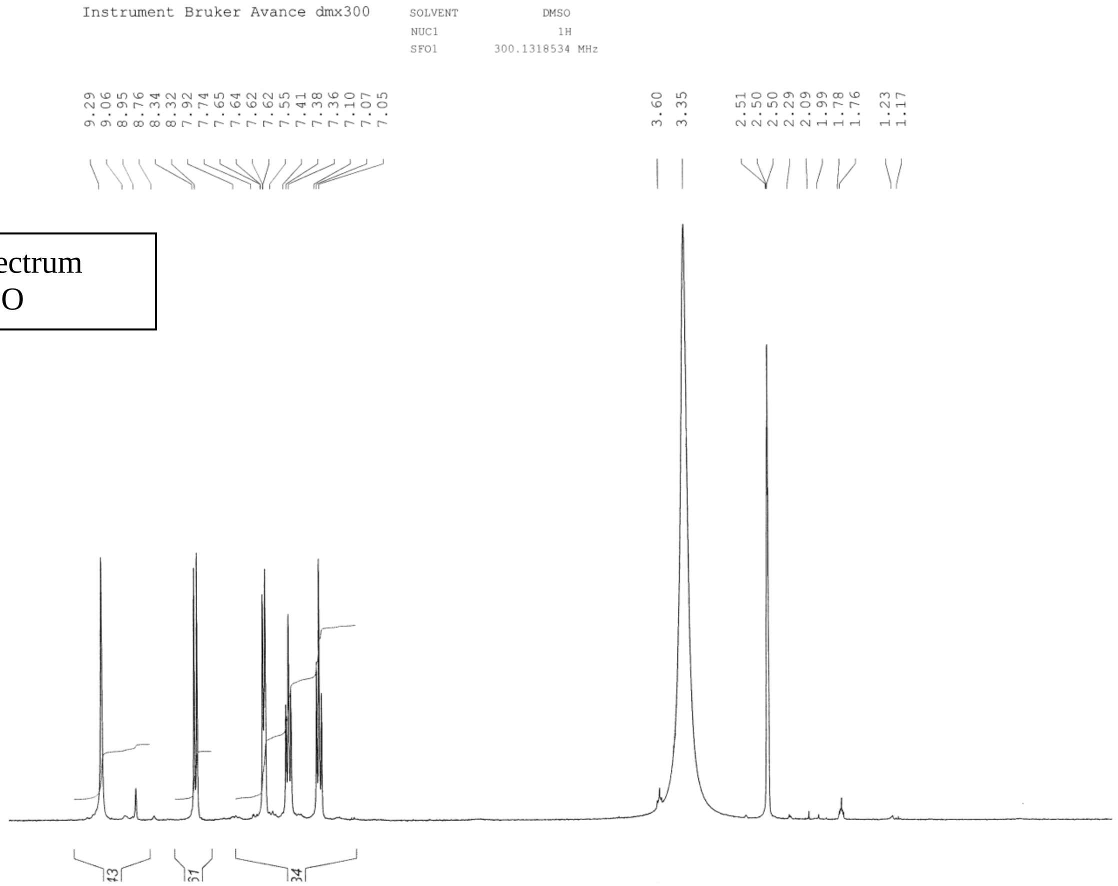

^{13}C-NMR spectrum of **1** in DMSO

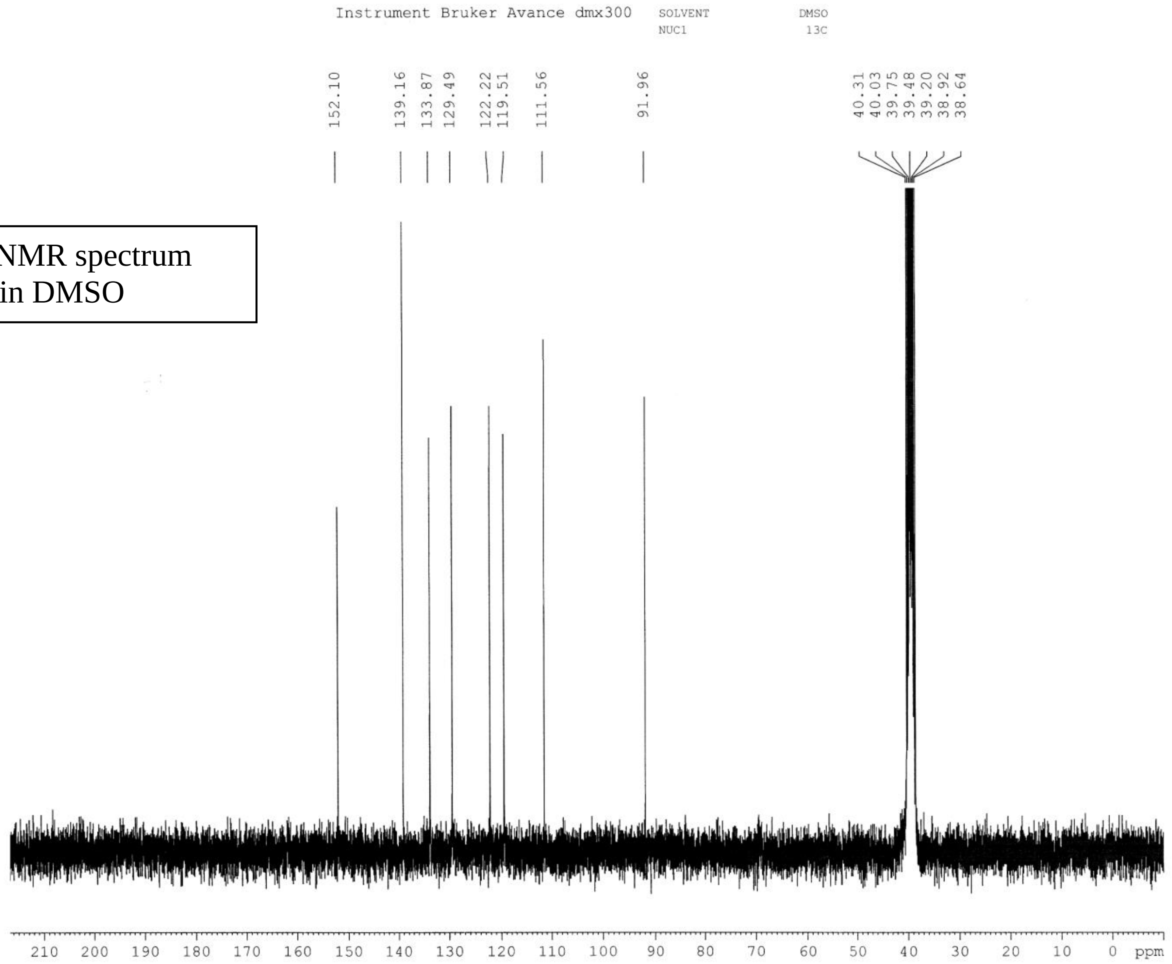

2. Synthesis of **3** by thermolysis of *cyclo*-bis(ethynyldiphenylurea) (**1**)

100 mg (0.213 mmol) of **1** were placed in a glass ampoule of 12 cm length and 1 cm internal diameter, which was evacuated to a final pressure of 10^{-3} mbar and closed by flame-sealing. The ampoule was placed in a horizontal aligned tube furnace in a temperature gradient. The side loaded with **1** was placed at 130 ^{o}C in the hot zone, the cold zone was kept at about 50 ^{o}C. Within one week, colourless crystals of **3** appeared in the cold zone of the ampoule. The yield and conversion reached 80 %. 80 mg (0.34 mmol) of **3** could be isolated. Some black tar remained in hot zone of the ampoule

Mass spectrum (EI, inlet temperature 100 ºC, *m*/*z* (Int.)): 234.1 (12) $[M]^{+}$; 208.1 (100); 180.1 (20); 152.0 (10); 104.0 (9), 89.0(6), 77.0 (4)

3. Synthesis of *cyclo*-bis(butadiynyldiphenylurea) (**2**)

a) Synthesis of 1,4-bis(2-aminophenyl)buta-1,3-diyne

The synthesis was performed according to C. Koradin, W. Dohle, A. L Rodriguez, B. Schmid, P.Knochel, *Tetrahedron* **2003**, *59*, 1571-1587.

b) Synthesis of **2**

720 mg (3.1 mmol, 1 equiv.) 1,4-bis(2-aminophenyl)buta-1,3-diyne were placed in a 250 mL flask and 70 mL of dry THF were added. After adding 1.5 g (9.30 mmol, 3 equiv.) of 1,1'-carbonyldiimidazole the mixture was heated under reflux for 7 h.
After cooling to room temperature the solvent was removed and the residue was mixed with ethyl acetate. The organic layer was washed with diluted hydrochloric acid and demineralised water. Drying over sodium sulphate, filtration and removing the solvent gave the desired product as a light yellow powder. Yield 480 mg (60 %).

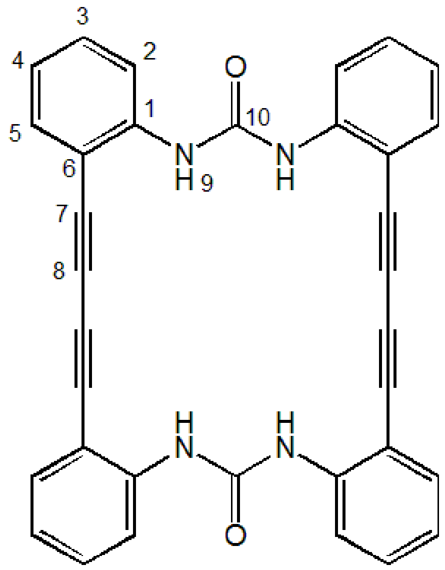

Formula and atom numbering of the cyclic bis(butadiynyldiphenylurea) **2**

^{1}H-NMR (pyridine-d_5) δ /ppm: 9.69 (s, 1H, NH); 8.79 (dd, 1H, CH(2), $^3J_{HH}$ = 8.20 Hz, $^1J_{HH}$ = 1.35 Hz); 7.44 – 7.53 (m, 2H, CH(3,5)); 7,04 (dt, 1H, CH(4), $^3J_{HH}$ = 7,59 Hz; $^1J_{HH}$ = 1.11 Hz)

^{13}C-NMR (pyridine-d_5) δ /ppm: 153.47 (C10); 142.38 (C1); 134.45 (C5); 131.35 (C3); 123.73 (C4); 122.20 (C2); 112.07 (C6); 80.59 (C7,C≡C); 79.92 (C8, C≡C)

Mass spectrum (EI, inlet temperature 280 °C, *m/z* (Int.)): 516.2 (48) $[M]^+$; 474.1 (47) $[M\text{-}NHCO]^+$, 284.1 (64) $[M\text{-}C_{15}H_9N_2O]^+$; 258.1 (100) $[M/2]^+$; 229.1 (70) $[C_{16}H_{10}N_2]^+$; 204,1 (28) $[C_{14}H_8N_2]^+$; 176.1 (10); 151.0 (9)

Mp: **2** turns dark blue above 200 °C, melts under decomposition >320 °C

Analysis: $C_{34}H_{20}N_4O_2$ (M_r = 516,54 g/mol) calc. 79.10 C, 3.90 H, 10.90 N; found when dried *in vacuo* at room temperature 76.70 C, 4.70 H, 10.4 N; found after heating *in vacuo* above 200 °C in the “blue state” 77.90 C, 4.80 H, 10.90 N.

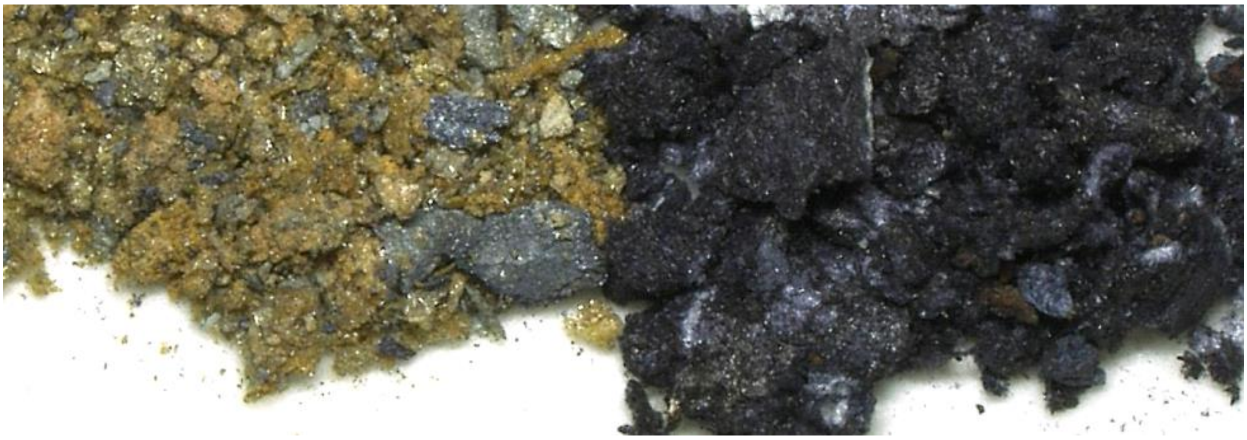

Fig. S2 Photographs of *cyclo*-bis(butadiynyldiphenylurea) **2**

The compound **2** has mainly a yellow appearance. When dried *in vacuo* at room temperature some of the material obtains a bluish colour (left). When dried *in vacuo* under application of elevated temperatures of higher than 100 ^{o}C the colour of the compound turns gradually to dark-blue (right). Spectroscopic investigations show no significant differences between the two “forms” (see text).

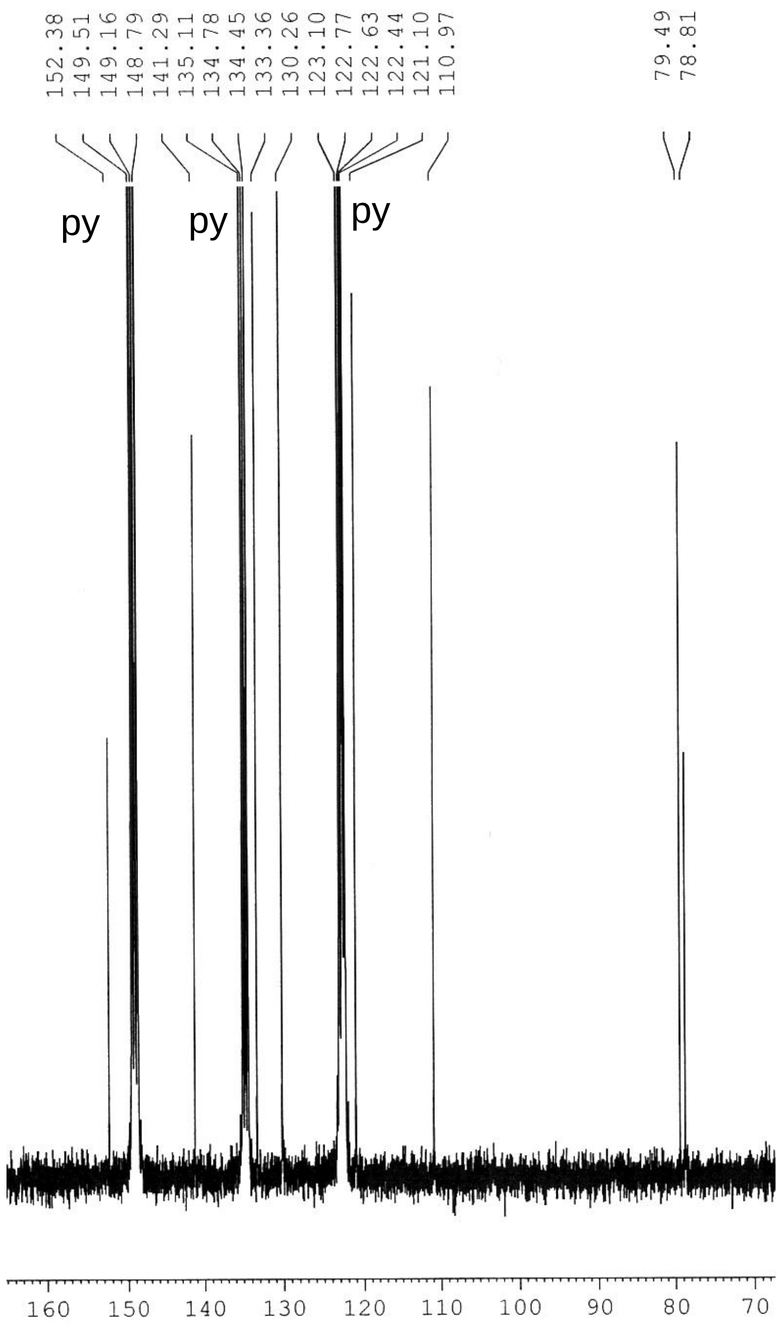

Fig. S3 ^{13}C-NMR spectrum of **2** in pyridine-d_5

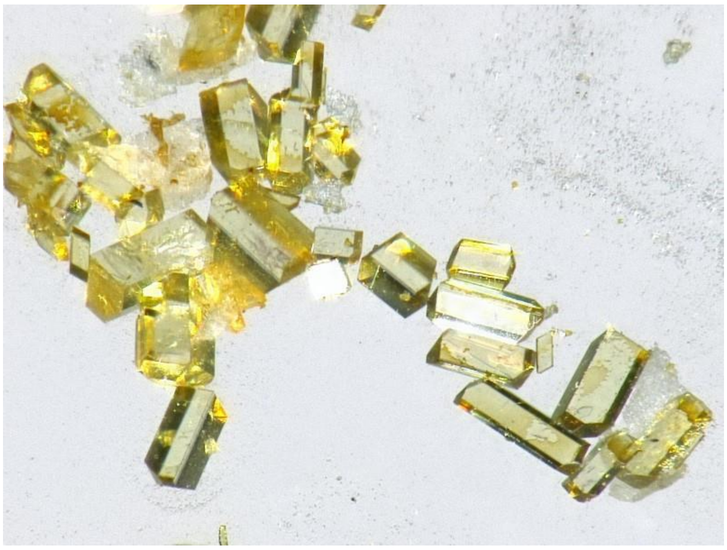

Fig. S4 Crystals of **2** · 2 DMSO (size approx. 0.5 mm)

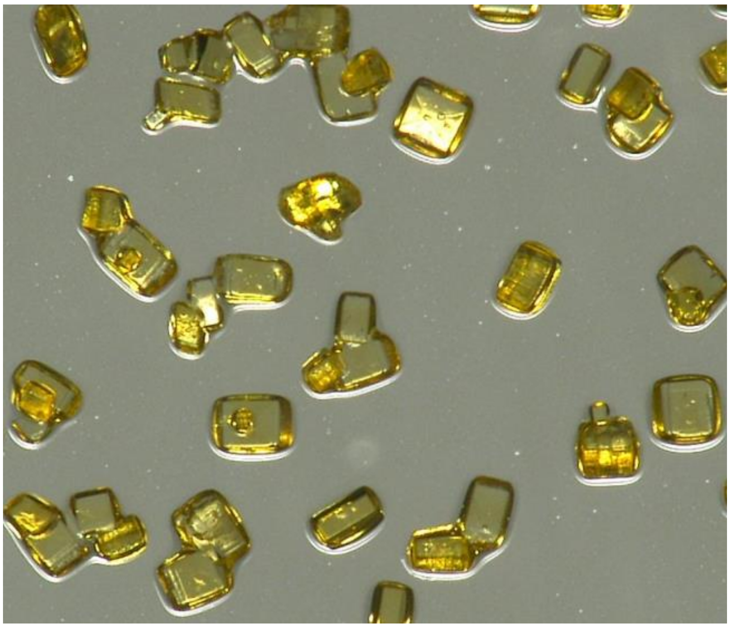

Fig. S5 Crystals of NEt_4[Br · **2**] (size approx. 0.4 mm)

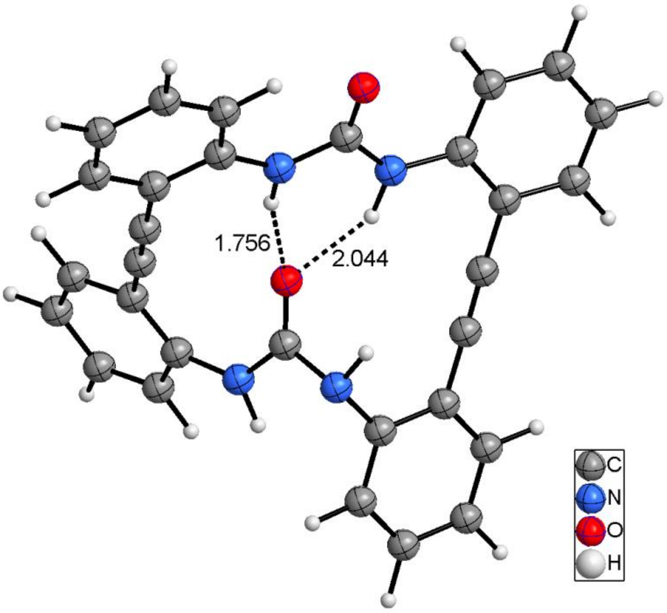

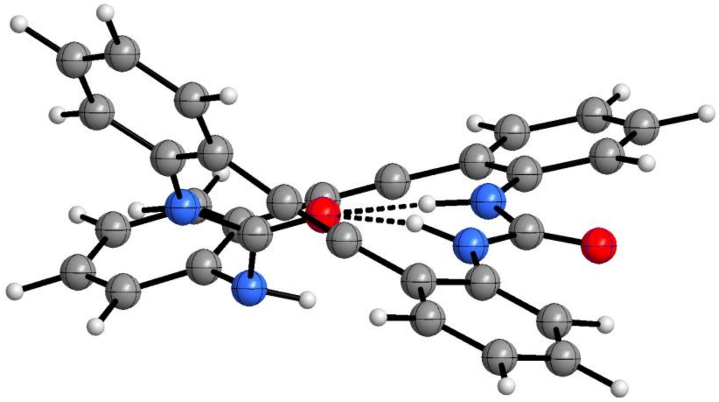

Fig. S6 The molecular structure of *cyclo*-bis(ethynyldiphenylurea) **1** from two different points of view, calculated by molecular mechanics using SPARTAN [1]. According to this calculation, the molecule is strongly tilted and deviates substantially from planarity. Strong intramolecular hydrogen bridges are present due to a head-to-tail arrangement of the two urea groups. The respective O···H distances are given in Å.

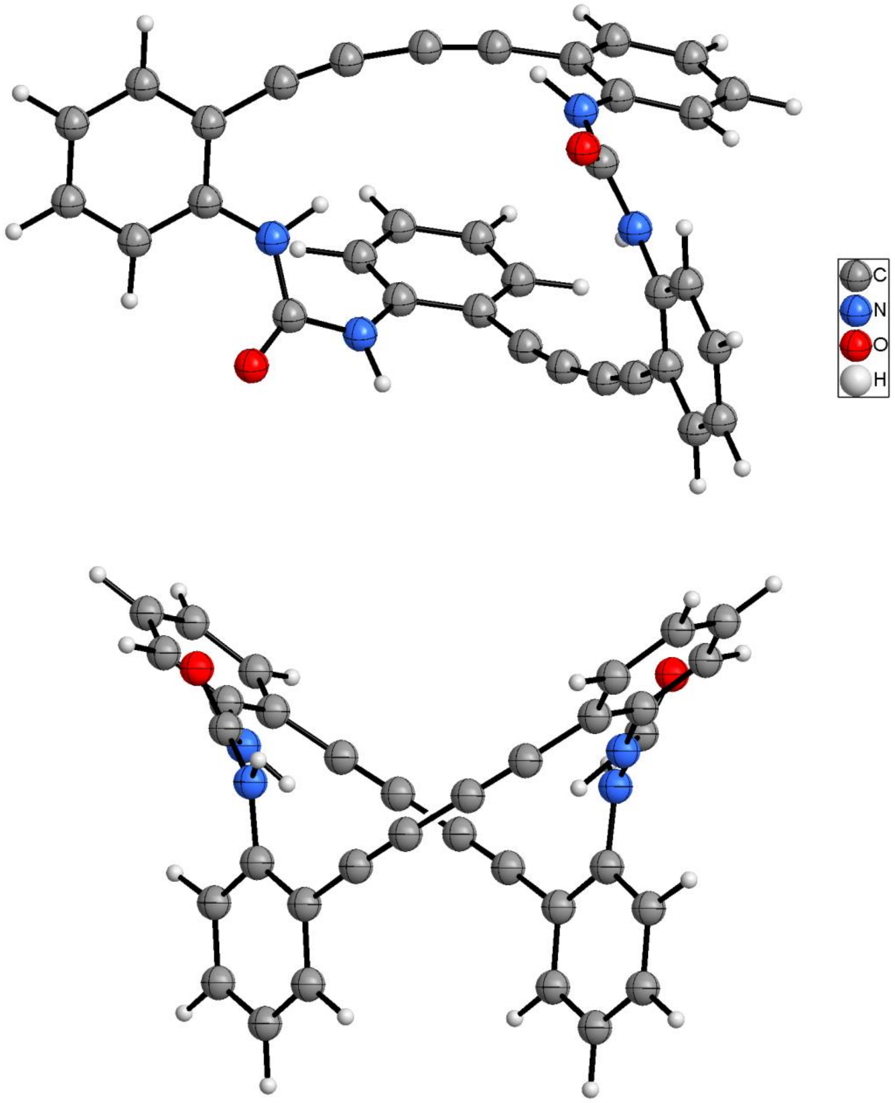

Fig. S7 The molecular structure of cyclo-bis(butadiynyldiphenylurea) **2** from two different points of view, calculated by molecular mechanics using SPARTAN [1]. According to this calculation no intramolecular hydrogen bridges are present. The molecule is strongly tilted.

[1] SPARTAN'10, Program for Molecular Modelling, *Wavefunction Inc.*, Irvine, CA, USA

X-Ray Crystal Structure Determinations

Crystals of compound **3** were obtained by sublimation, crystals of **2** · 2 DMF, **2** · 2 DMSO and NEt_4[Br · **2**] by slow evaporation of the respective solutions. Diffraction data sets were recorded using a Nonius MACH3 diffractometer for **3** and **2** · 2 DMF, a Stoe IPDS diffractometer for **2** · 2 DMSO and a Bruker κ-CCD diffractometer for NEt_4[Br · **2**], all equipped with graphite monochromatized Mo-Kα radiation. Intensities were measured at room temperature for **3**, **2** · 2 DMF, NEt_4[Br · **2**] and for **2** · 2 DMSO under cooling to 123 K. Crystal systems, lattice types and the space groups were derived from simulated precession photographs. The crystal structures were solved by direct methods [2] and refined based on full-matrix least-squares methods on F^2 with the SHELX-97 programs.[3] All non-hydrogen atoms were refined with anisotropic displacement parameters, all hydrogen atoms with free positional parameters and isotropic displacement parameters. The sulphur atom of the DMSO molecule in the structure of **2** · 2 DMSO turned out as disordered over two positions. The occupation factors were refined to 0.869(3) for site S1A and 0.131(3) for site S1B.

X-Ray crystallographic data and refinement details for all four structure determinations are summarized in Table S1.

Further details of the crystal structure investigations have been deposited with the Cambridge Crystallographic Data Centre. The data in cif format may be obtained free of charge via http://www.ccdc.cam.ac.uk/ on quoting the deposit numbers CCDC 993209 (**2** · 2 DMF), CCDC 993210 (**2** · 2 DMSO), CCDC 993207 (NEt_4[Br · **2**]), CCDC 993208 (**3**).

[2] G. M. Sheldrick, *SHELXS97*, Program for Crystal Structure Solution, University of Göttingen, Germany, **1997**.

[3] G. M. Sheldrick, *SHELXL97*, Program for Crystal Structure Refinement, University of Göttingen, Germany, **1997**.

Table S1. Crystallographic data and details of crystal structure refinement for the compounds **3**, **2** · 2 DMF, **2** · 2 DMSO and NEt_4[Br • **2**]. Numbers in parentheses are standard deviations and refer to the last significant digit.

Compound	**3**	**2** · 2 DMF	**2** · 2 DMSO	NEt_4[Br · **2**]
Formula	$C_{15}H_{10}N_2O$	$C_{40}H_{34}N_6O_4$	$C_{38}H_{32}N_4O_4S_2$	$C_{42}H_{40}BrN_5O_2$
M_r / g mol^{-1}	234.25	662.74	672.82	726.70
Crystal system	monoclinic	triclinic	monoclinic	monoclinic
Space group	$P2_1/c$	$P\bar{1}$	$P2_1/c$	$P2_1/c$
a /Å	11.830(2)	8.2597(9)	8.224(1)	12.7152(6)
b /Å	7.098(2)	9.772(1)	17.995(2)	17.5556(8)
c /Å	13.470(1)	11.023(1)	12.120(2)	20.5472(8)
α / °		95.851(7)		
β / °	102.437(8)	99.783(8)	113.48(1)	125.548(3)
γ / °		100.761(9)		
V /Å^3	1104.5(3)	853.1(2)	1645.1(4)	3731.8(3)
Z	4	1	2	4
T / K	293(2)	293(2)	123(2)	293(2)
ρ / g cm^{-3}	1.41	1.29	1.358	1.29
μ / mm^{-1}	0.09	0.09	0.21	1.14
F(000)	488	348	794	1512
Measured reflections	5487	6682	6855	32699
Independent reflections	2534	3341	3136	6837
R_{int}	0.049	0.025	0.105	0.143
Reflections with $I > 2\sigma(I)$	1449	2174	1261	3078
No. of parameters	203	295	227	535
$R(F)$ ($Fo > 4\sigma(Fo)$)	0.042	0.036	0.052	0.062
$R(F)$ (all data)	0.099	0.073	0.153	0.165
$wR(F^2)$	0.116	0.105	0.104	0.163
GooF	0.985	0.991	0.738	1.005
Residual electron density /e Å^{-3}	0.14 / -0.18	0.18 / -0.16	0.18 / -0.24	0.33 / -0.30

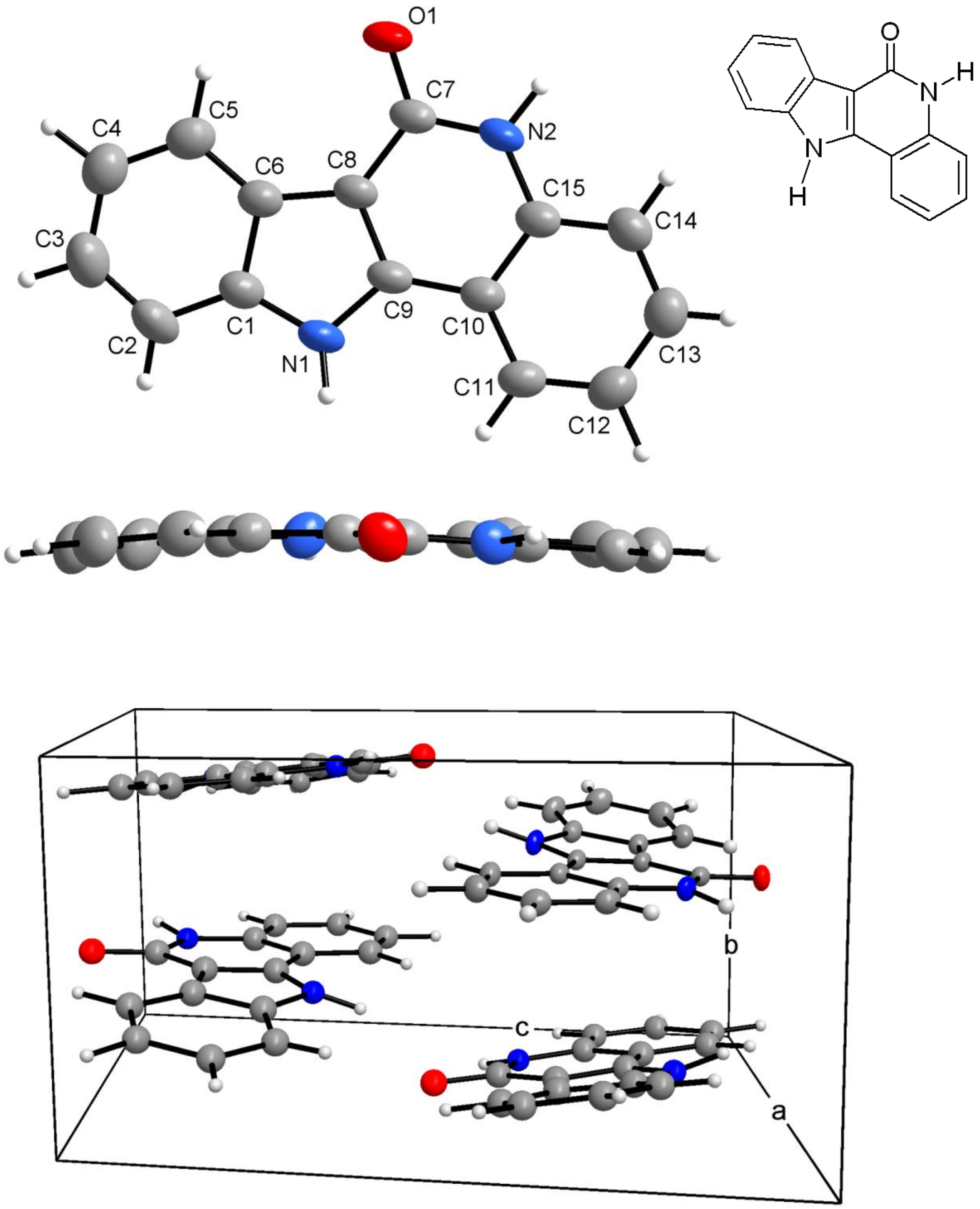

Fig. S8 The molecular and crystal structure of dihydroindoloquinolinone **3**. On top: view of the molecule given perpendicular to the molecular plane. Middle: view parallel to the molecular plane showing the almost complete planarity of the molecule. On bottom: view of the unit cell in a perspective view along the *a* axis showing the parallel arrangement of the molecules in the crystal. Displacement ellipsoids are drawn to include a probability density of 50 %. H atoms are drawn with arbitrary radii.

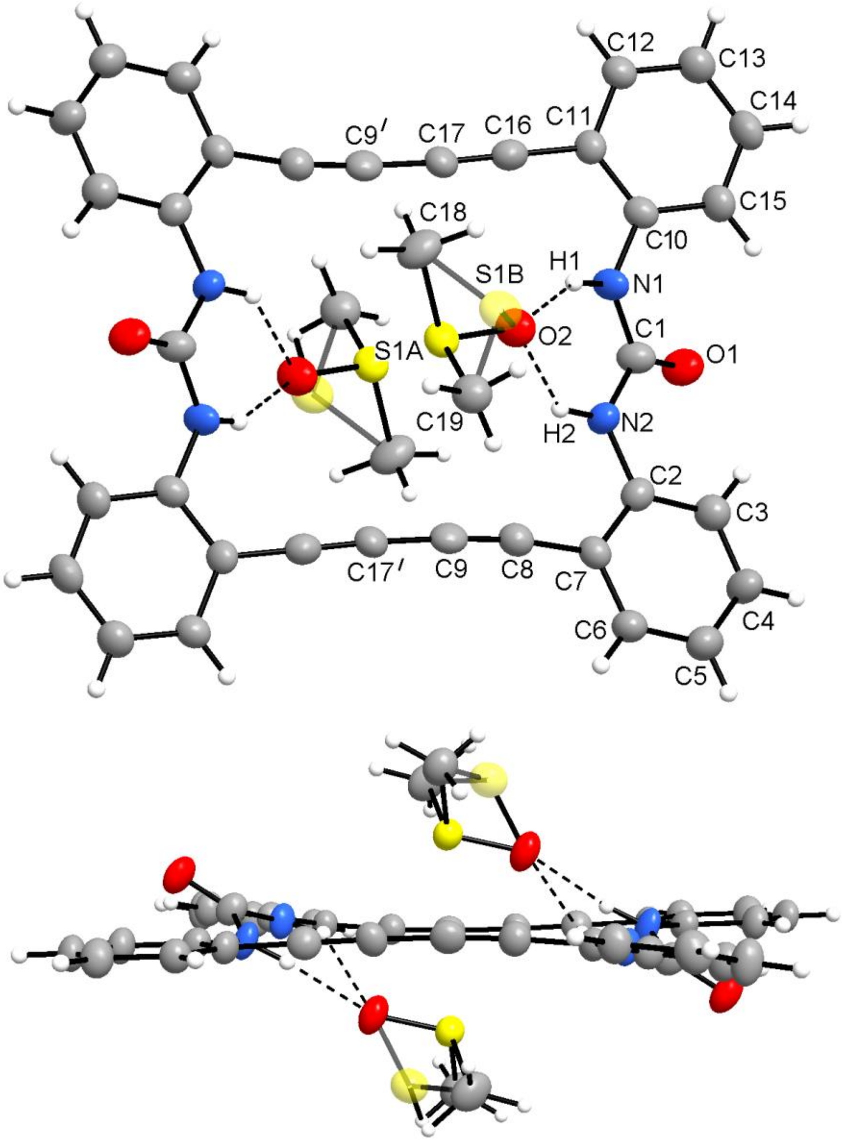

Fig. S9 The molecular structure of **2** · 2 DMSO in two different views, on top perpendicular to the plane, on bottom in the plane of the macrocyclus. The molecular complex bears an inversion centre in the midpoint. Thermal ellipsoids are scaled at the 50% probability level.

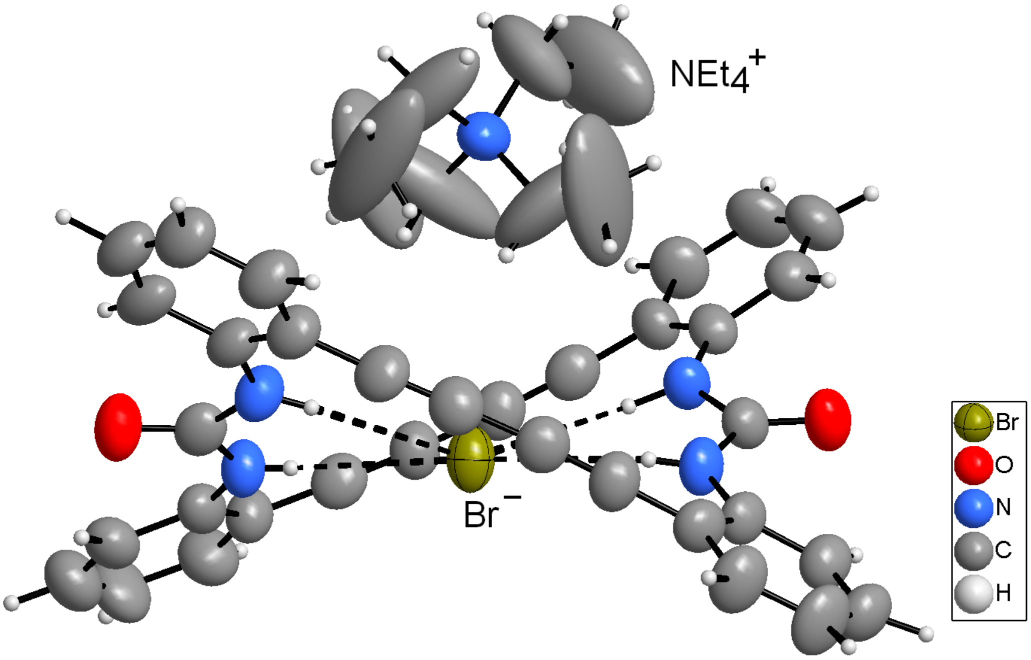

Fig. S10 The molecular structure of NEt_4[Br · **2**]. Thermal ellipsoids are scaled at the 50% probability level. The carbon atoms of the tetraethylammonium cation show large displacement ellipsoids, probably caused by some positional disorder and enhanced thermal vibration. Diffraction data collection was performed at ambient temperature.

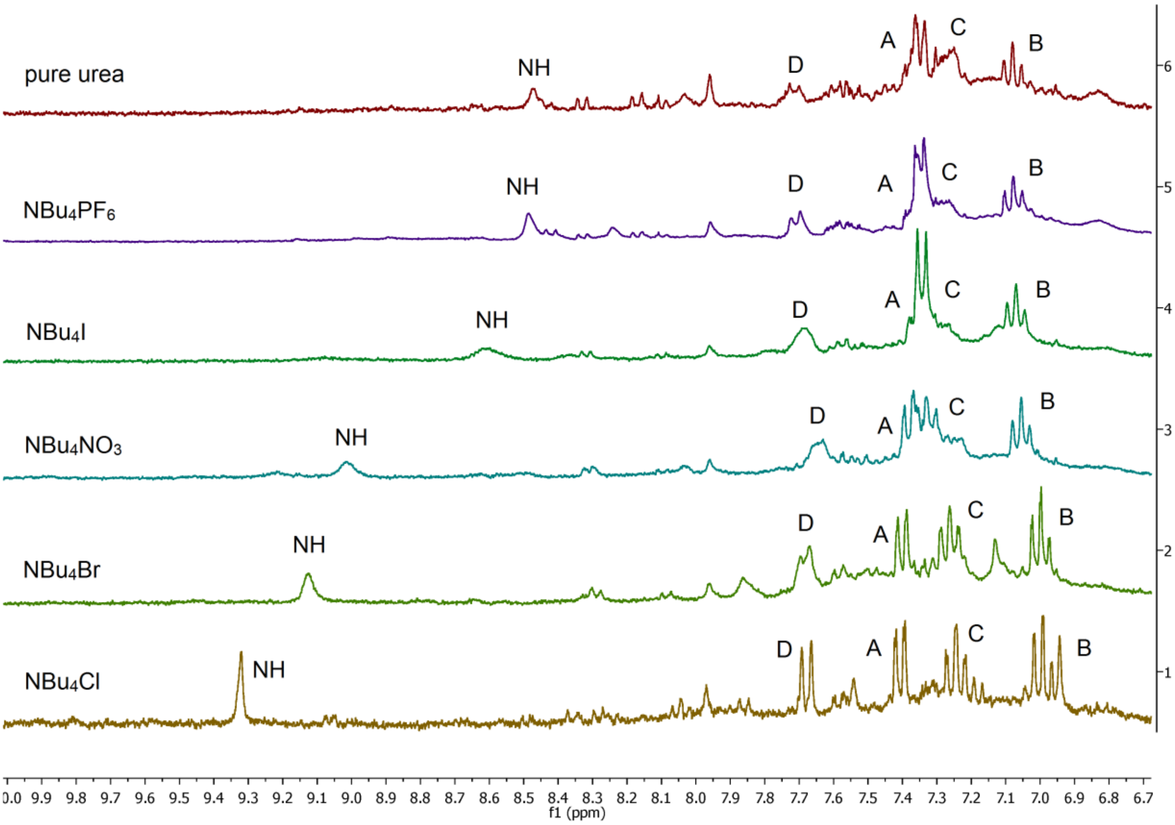

Fig. S11 ^{1}H-NMR spectra of **1** in acetone-D_6 after addition of several different tetrabutyl-ammonium salts. The N−H proton resonances show a difference in the chemical shifts of up to 0.9 ppm depending of the type of anion present.

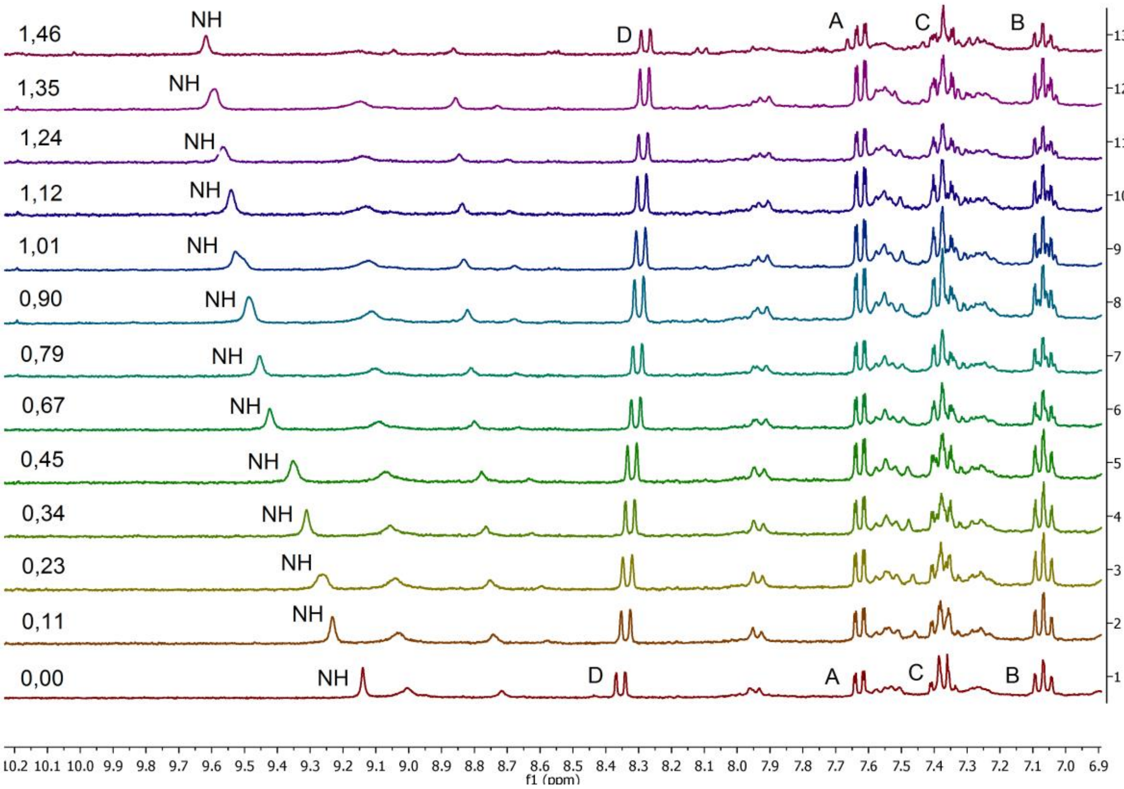

Fig. S12 ^{1}H-NMR spectra of **1** in DMSO-D_6 after addition of increasing molar equivalents of tetrabutylammonium chloride. The N−H proton resonance shows a slight downfield shift depending on the concentration of the anion.

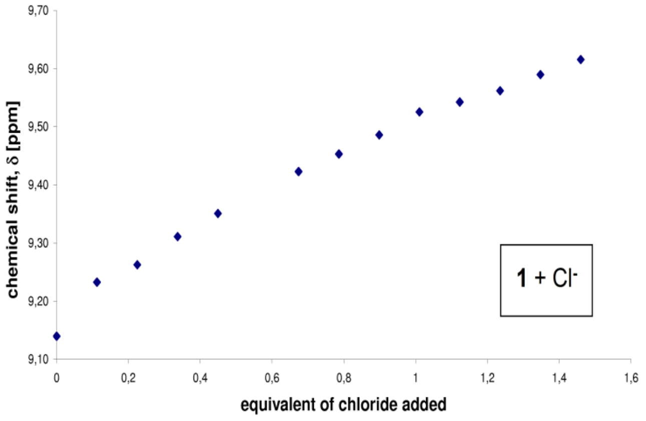

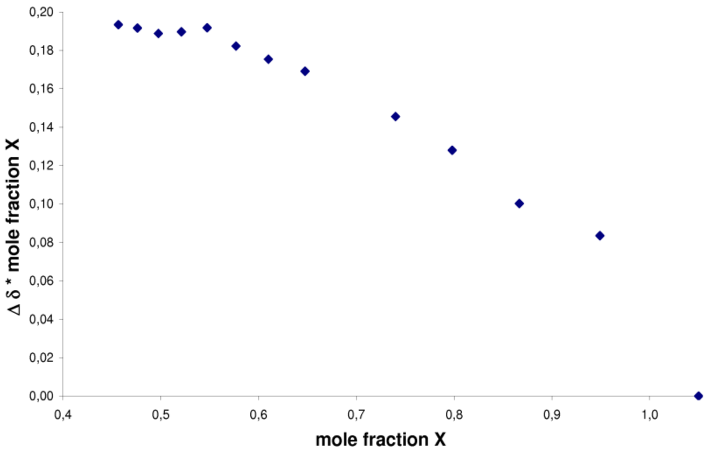

Fig. S13 Variation of the chemical shift of the N−H ^{1}H-NMR signal of **1** on addition of tetrabutylammonium chloride. On the bottom the respective Job plot is shown.

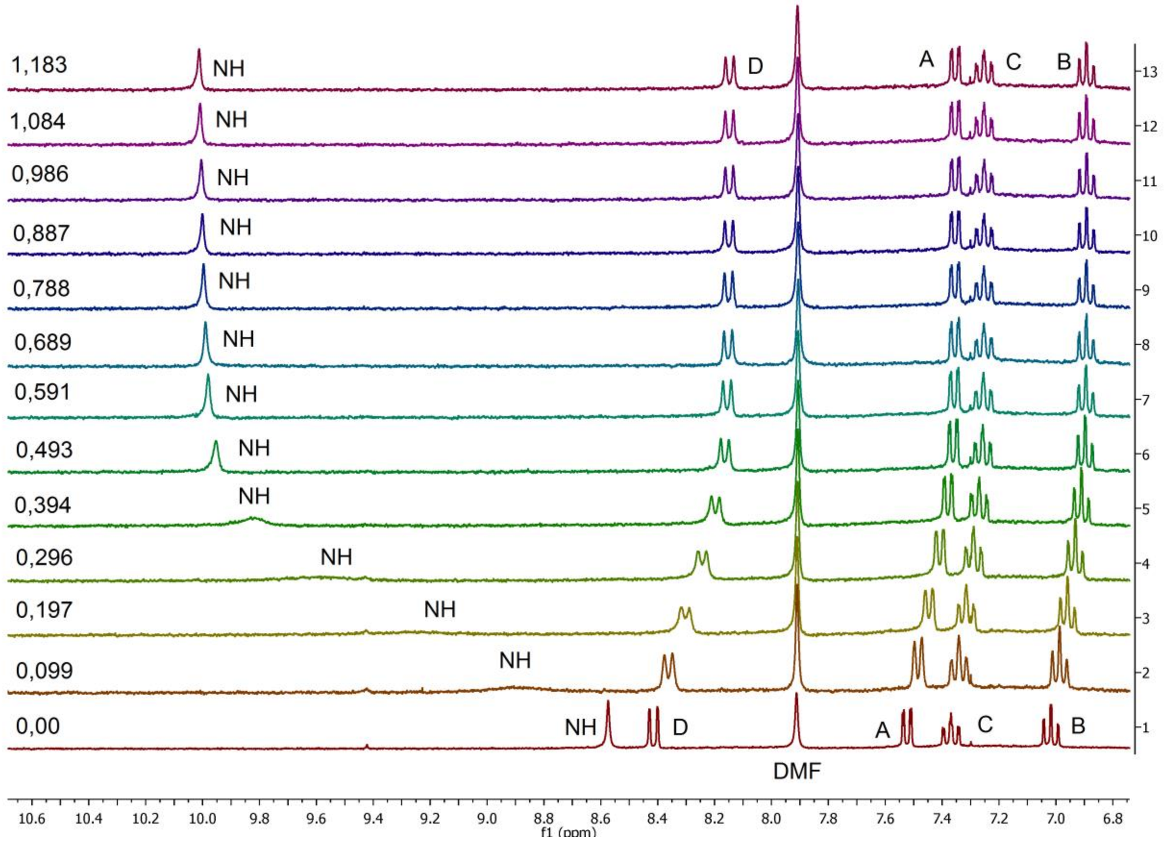

Fig. S14 ^{1}H-NMR spectra of **2** in THF-D_8 after addition of increasing molar equivalents of tetrabutylammonium chloride. The N−H proton resonance shows a distinct downfield shift dependent on the concentration of the anion. The small DMF signal originates from the solvent of recrystallization used to purify the samples for the NMR experiments.

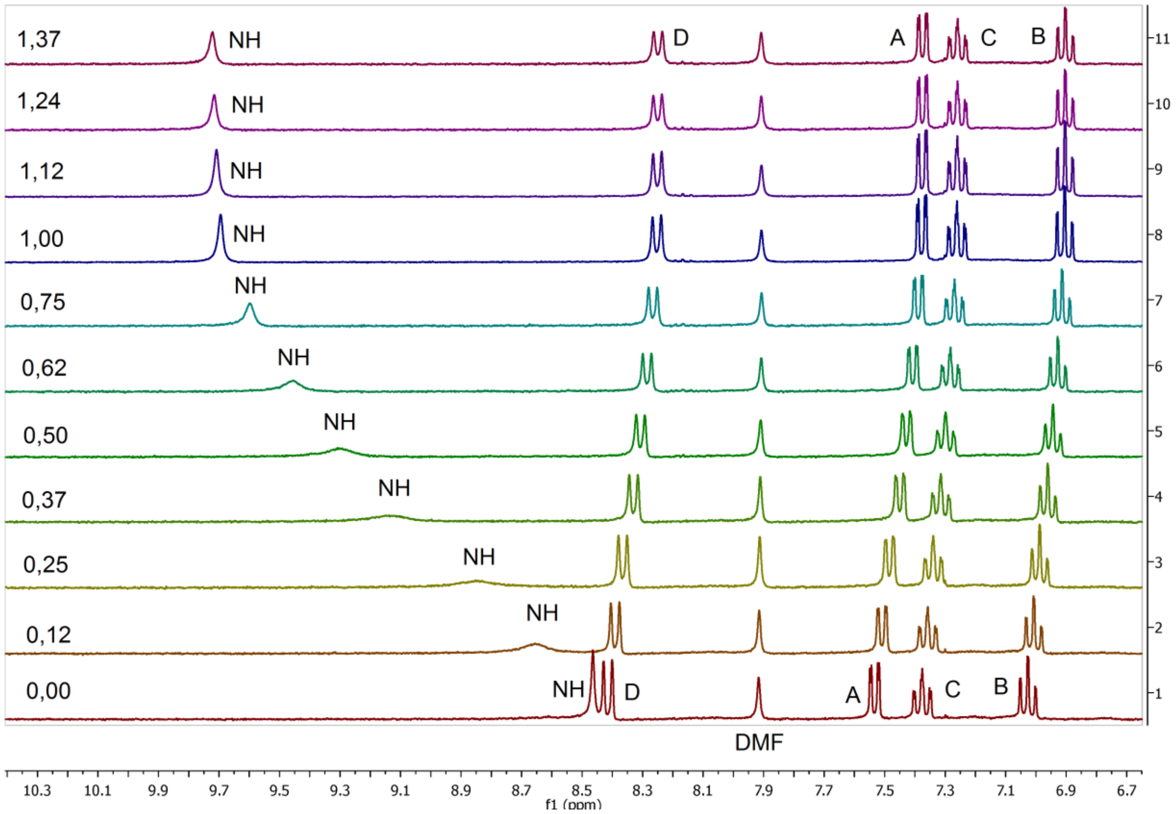

Fig. S15 ^{1}H-NMR spectra of **2** in THF-D_8 after addition of increasing molar equivalents of tetrabutylammonium bromide. The N−H proton resonance shows a distinct downfield shift dependent on the concentration of the anion. The small DMF signal originates from the solvent of recrystallization used to purify the samples for the NMR experiments.

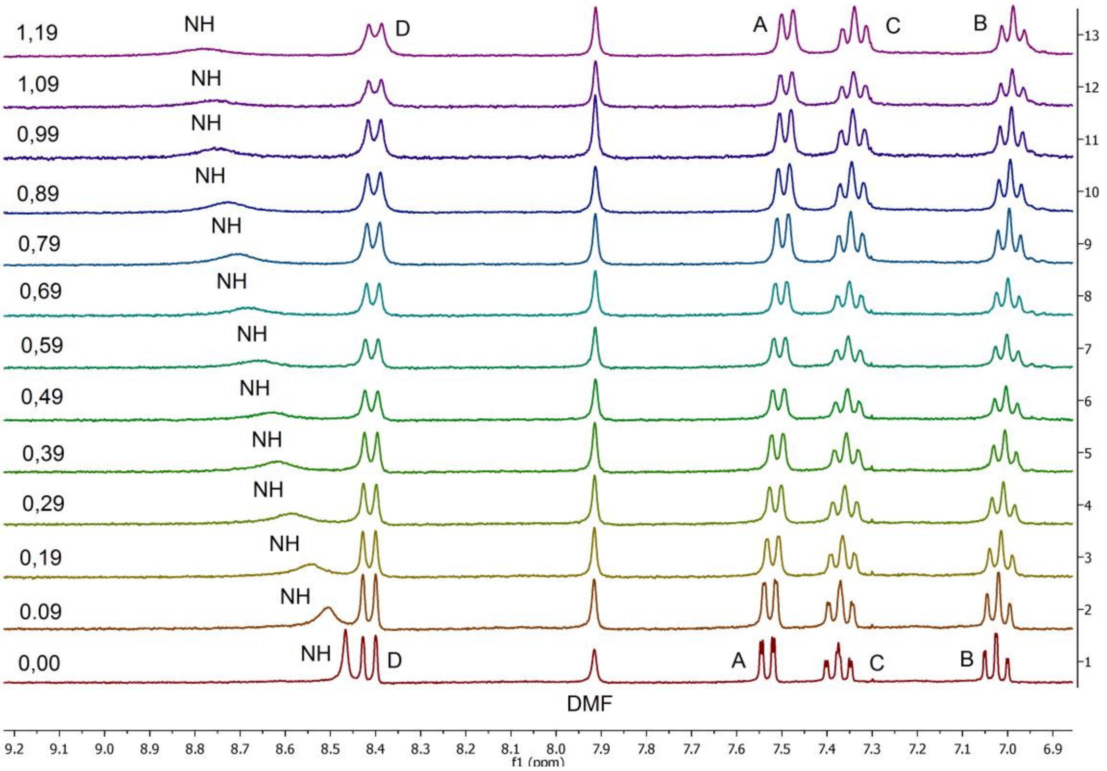

Fig. S16 ^{1}H-NMR spectra of **2** in THF-D_8 after addition of increasing molar equivalents of tetrabutylammonium iodide. The N−H proton resonance shows a slight downfield shift dependent on the concentration of the anion. The small DMF signal originates from the solvent of recrystallization used to purify the samples for the NMR experiments.

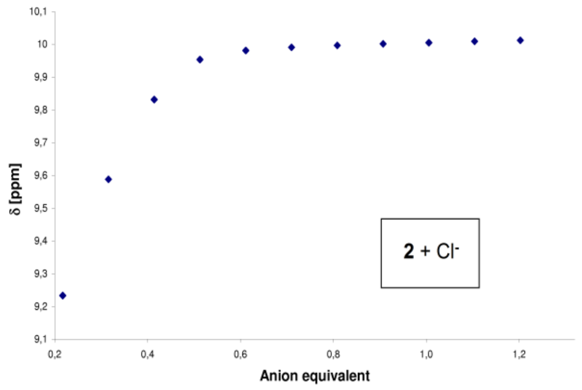
δ [ppm]
Anion equivalent
2 + Cl-

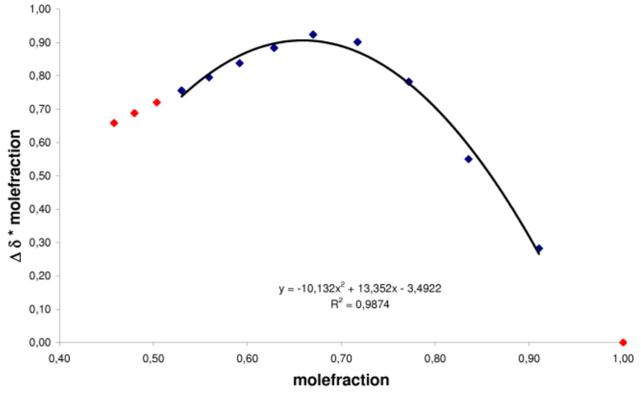
Δ δ * molefraction
molefraction
y = -10,132x² + 13,352x - 3,4922
R² = 0,9874

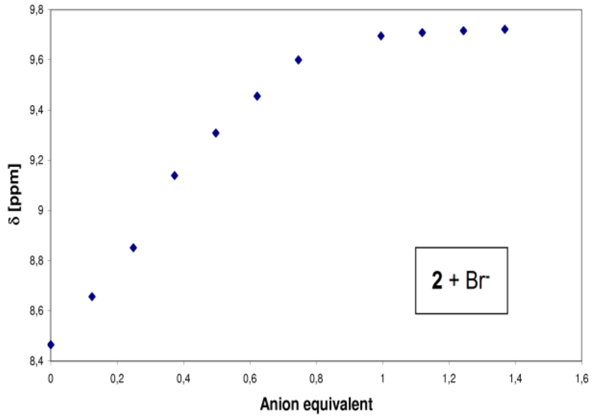
δ [ppm]
Anion equivalent
2 + Br-

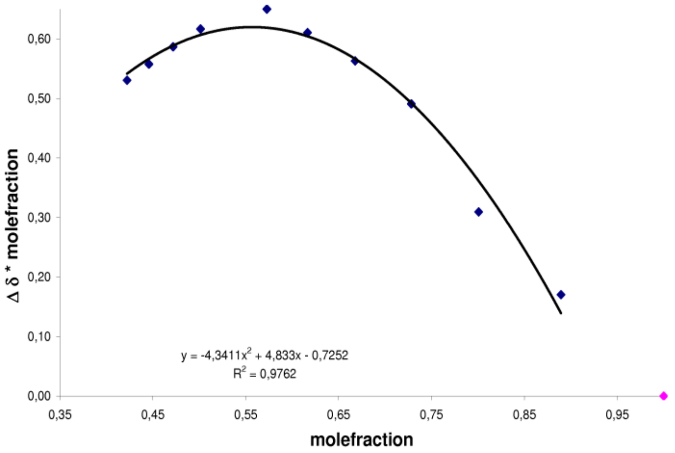
Δ δ * molefraction
molefraction
y = -4,3411x² + 4,833x - 0,7252
R² = 0,9762

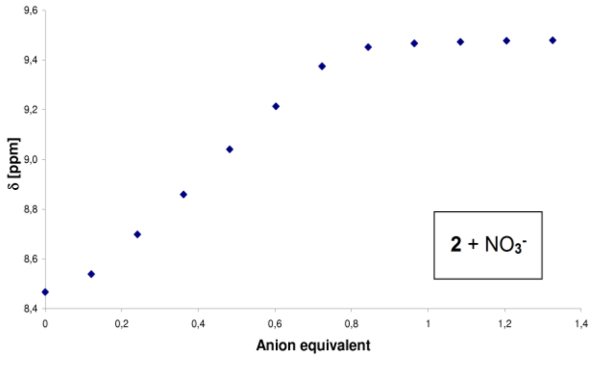
δ [ppm]
Anion equivalent
2 + NO3-

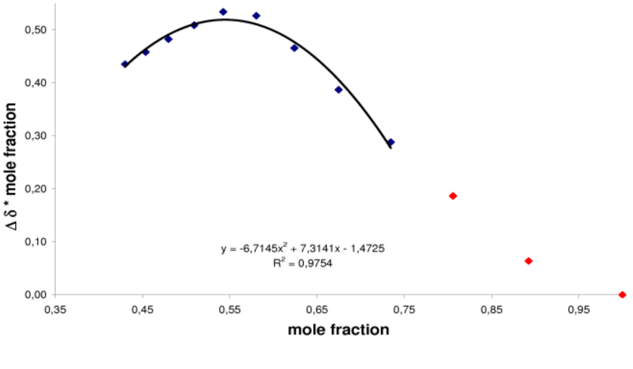
Δ δ * mole fraction
mole fraction
y = -6,7145x² + 7,3141x - 1,4725
R² = 0,9754

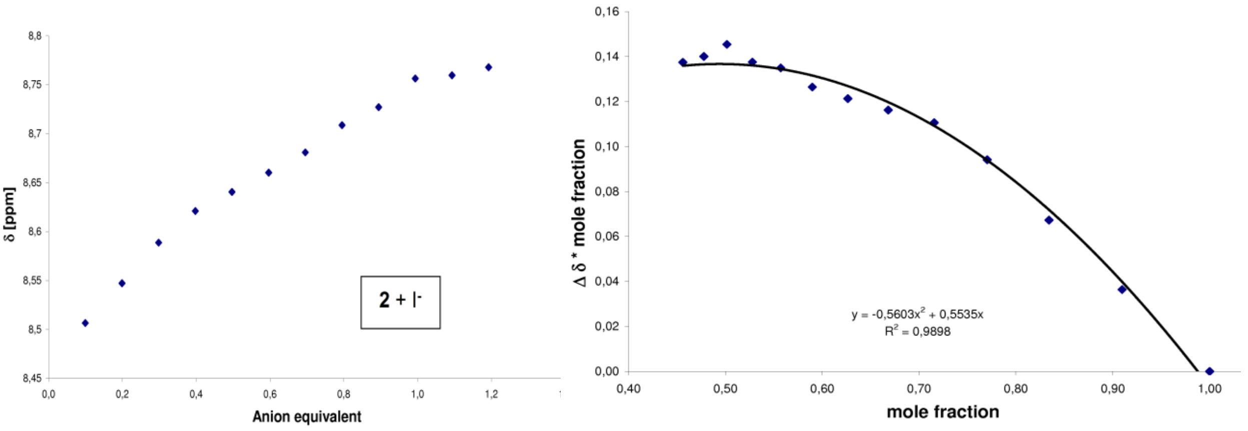

Fig. S17 Variation of the chemical shift of the N−H ^{1}H-NMR signal of **2** on addition of various anions as the respective tetrabutylammonium salts. On the right the respective Job plots are shown.
